# Information Dynamic Correlation of Vibration in Nonlinear Systems

**DOI:** 10.3390/e22010056

**Published:** 2019-12-31

**Authors:** Zhe Wu, Guang Yang, Qiang Zhang, Shengyue Tan, Shuyong Hou

**Affiliations:** 1 School of Mechanical Engineering, Hebei University of Science and Technology, Shijiazhuang 050018, China; tanshengyue@gmail.com (S.T.); shuyonghou@gmail.com (S.H.); 2 School of Mechanical and Vehicle Engineering, Beijing Institute of Technology, Beijing 100081, China; qiangzh36@gmail.com; 3 Key Laboratory of Vehicle Transmission, China North Vehicle Research Institute, Beijing 100072, China

**Keywords:** information dynamics correlation, Von Neumann entropy, nonlinear system, Rényi entropy, full vector multi-scale Rényi entropy

## Abstract

In previous studies, information dynamics methods such as Von Neumann entropy and Rényi entropy played an important role in many fields, covering both macroscopic and microscopic studies. They have a solid theoretical foundation, but there are few reports in the field of mechanical nonlinear systems. So, can we apply Von Neumann entropy and Rényi entropy to study and analyze the dynamic behavior of macroscopic nonlinear systems? In view of the current lack of suitable methods to characterize the dynamics behavior of mechanical systems from the perspective of nonlinear system correlation, we propose a new method to describe the nonlinear features and coupling relationship of mechanical systems. This manuscript verifies the above hypothesis by using a typical chaotic system and a real macroscopic physical nonlinear system through theory and practical methods. The nonlinear vibration correlation in multi-body mechanical systems is very complex. We propose a full-vector multi-scale Rényi entropy for exploring the chaos and correlation between the dynamic behaviors of mechanical nonlinear systems. The research results prove the effectiveness of the proposed method in modal identification, system dynamics evolution and fault diagnosis of nonlinear systems. It is of great significance to extend these studies to the field of mechanical nonlinear system dynamics.

## 1. Introduction

Quantum entanglement is one of the most interesting properties in quantum mechanics [1,2,3]. It describes the whole properties of the entangled system. In a quantum entangled system, the correlation between the properties of the subsystems is not separable in the space domain. As long as there is entanglement between the subsystems, no matter how far away they are, there is still interaction between them. Because of the coherence and entanglement between quantum systems, there are a lot of available information resources in the entangled system, therefore, it is possible for information transmission to cross the space limit. Quantum entanglement plays an important role in quantum computation [4], quantum information processing [5] and quantum physical systems [6,7,8]. The measurement of the correlation between physical systems is the core of the research on complex physical systems under the background of modern technology [9], but the correlation between physical systems cannot be explained by classical physics [4,5]. In physics, entropy can be interpreted as the measurement of the disorder degree of the system [3]. It can measure the uncertainty of the state of a physical system [9,10]. Methods such as Von Neumann entropy, relative entropy, Rényi entropy and linear entropy can measure the complexity of nonlinear systems [11,12,13,14,15,16,17,18,19]. Researchers have used Von Neumann entropy and Rényi entropy and other information dynamics methods to conduct in-depth research [16,17,18] in the fields of quantum information transmission [19,20,21], molecular dynamics systems [22] and superconducting circuits [23,24].

For the non-entangled states, the mixed state can be decomposed into the product states of all pure state subsystems, and the subsystems appear in the composite system with a certain probability. At this time, the properties of other subsystems will not work, and the entropy can be used to measure and quantify the system [25]. In a series of recent pioneering works, the application of information dynamics methods such as Von Neumann entropy and Rényi entropy in microcosmic nonlinear dynamics system is discussed. When the literature [26,27,28,29,30] quantifies the various behaviors of entanglement by Von Neumann entropy or linear entropy [31], it is proved that nonlinear systems interactions can be characterized by entanglement entropy. Vedral [32] discusses in depth the entanglement measure and application of Von Neumann Reduced Entropy and Relative Entanglement Entropy. Feng et al. [33] used algebraic methods to explore the entanglement dynamics of the non-harmonic vibration of real molecules in triatomic molecules. Abdel-Aty et al. [34] comprehensively analyzed the information entropy pattern generated during the time evolution of the interaction between ions and laser fields, and established a clear relationship between precise information entropy and multi-level ions and laser fields. Hou et al. [35] uses the reduced density linear entropy method to study the dynamic entanglement of two kinds of stretching vibrations of triatomic molecules H_2_O and SO_2_ in algebraic models under different MQN and initial states, so that different entanglement behaviors in these two molecules can be characterized. Liu et al. [3] used an algebraic model to study the dynamics entanglement of small molecule vibrations, and gave the analytical expressions of linear entropy, Von Neumann entropy and Lyapunov function of the integrable dimer and the actual small molecule in the initial Fock state and coherent state. Kis et al. [36] uses analytical methods to determine the vibrational state of polyatomic molecules excited by the optically limited pulse, and uses Von Neumann entropy to describe the size and vibration mode of the entanglement. Ecker et al. [37] used holography to numerically study the entanglement entropy and quantum zero energy conditions in strongly coupled far non-equilibrium quantum states. Wang et al. [38] studied quantum entanglement in two-dimensional ion trap systems. The quantum entanglement between ions and phonons is discussed by using the Reduced Entropy, and the quantum entanglement between the two degrees of freedom of the vibrational motion in the *x* and *y* directions is discussed by using the quantum Relative Entropy. Yuan et al. [15] studied the quantum entropy, energy and entanglement dynamics of different initial states in an important spectral Hamiltonian of the curved triatomic molecules H_2_O, D_2_O and H_2_S. 

The information dynamics methods such as Von Neumann entropy and Rényi entropy have experienced the development from theory to experiment, from microcosmic to macroscopic, from discrete to continuous [39,40,41,42,43]. In all of these studies, people prefer and focus on the entanglement of microscopic physical systems. There are few studies on macroscopic physical systems. There are complex connections and differences between microscopic quantum signals and macroscopic physical signals. We use Von Neumann entropy and Rényi entropy to explore the modal identification, system dynamics evolution and fault diagnosis of nonlinear mechanical system. It is of great significance to extend these studies to the study of nonlinear system dynamics.

Rotating Machinery Structural Health Monitoring (SHM) is an important means to identify potential faults, evaluate operation status and predict reliable operation probability based on collected condition monitoring data [44,45]. In the case of structural health degradation, the corresponding vibration signal will exhibit a change caused by a potential failure [46,47,48,49].

In large-scale rotating machinery transmission, due to the occurrence of sporadic failure of key components to stimulate the nonlinear vibration of the entire mechanical system, the vibration signal of the health monitoring of the rotating mechanical structure has many typical characteristics, which can be roughly divided into two categories: (1) Internal characteristics: strong nonlinearity, instability, strong coupling, etc. (2) External characteristics of the signal: external complex excitation and noise have obvious influence on system vibration. The internal and external characteristics of the signal interact and couple with each other. We try to use the Von Neumann entropy and Rényi entropy to analyze the strong nonlinearity, instability, strong coupling and other characteristics of the signal, extract the sensitive characteristic variables of the signal, and perform modal identification. Then the second problem we face next is how to reduce the interference of complex external excitation and improve the signal-to-noise ratio to some extent, because noise has a great influence on the Von Neumann entropy and Rényi entropy between the calculated signals. Therefore, robust data denoising technology must be developed to keep the features sensitive to changes of interest [50]. The large amount of data and low signal-to-noise ratio increase the difficulty of vibration signal processing. Therefore, research on how to solve the problem of excessive data volume during state monitoring and how to effectively reduce noise in data has attracted researchers in various fields [51].

As a new sampling theory, compressed sensing obtains discrete samples of signals by studying the sparse characteristics of signals, and perfectly reconstructs signals by nonlinear reconstruction algorithm [52,53,54]. Compressed sensing technology can be used to reconstruct sparse signals with noisy interference and no-noise interference [55], and has strong robustness and sparsity for multi-level quantization of measurement data [56]. Once proposed, it has received great attention in the fields of information theory [57], image processing [58,59,60], radar imaging [61,62], and biomedical engineering [63,64].

Changes in the environment in which the machine operates are unpredictable [65]. When critical transmission components of large mechanical equipment, such as planetary gears and bearings, fail, their vibration signals have typical strong nonlinear characteristics. Condition monitoring, fault diagnosis and dynamic inversion of mechanical equipment have always been the hotspots and difficulties of scholars in related fields in various countries. Therefore, it is very suitable to use the chaotic system and the real rotating mechanical nonlinear system to verify the validity, superiority and universality of the Von Neumann entropy and Rényi entropy theory. The nonlinear vibration correlation in multi-body mechanical systems is very complicated. Therefore, it is very meaningful and necessary to study the relationship between Von Neumann entropy and Rényi entropy and vibration interaction in nonlinear systems.

The main contribution of this work is outlined as follows:

(1) By establishing the state density matrix of nonlinear mechanical systems, the state characteristics of nonlinear mechanical systems and Lü’s chaotic systems are described. The full-vector multi-scale Rényi entropy based on homology information fusion is constructed. A method is proposed to quanlifies the degree of chaos, nonlinear characteristics and coupling relationship of the system by using Von Neumann entropy and full vector multi-scale Rényi entropy. Von Neumann entropy and Rényi entropy are successfully applied to the field of mechanical system dynamics.
(2) By using Von Neumann entropy and Rényi entropy, the chaotic degree, nonlinear characteristics and coupling relationship of Lü’s chaotic system and nonlinear mechanical system can be quantified, so as to achieve the purpose of mode identification, system time evolution and fault diagnosis.
(3) In the study, we found some rules between Rényi entropy and its scale parameters.

The outline of the manuscript is as follows: In Section 2, we describe in detail the theory and properties of Von Neumann entropy and Rényi entropy. At the same time, the theory of compressed sensing is introduced, and the noise reduction characteristics of compressed sensing are deeply studied. In Section 3, we simulate the time evolution of chaotic systems, and use the Von Neumann entropy and Rényi entropy to analyze the chaotic characteristics and coupling strength between the causal information of Lü’s chaotic systems, and compare their dynamic behaviors in different initial states. In Section 4, Von Neumann entropy and Rényi entropy is applied to the typical nonlinear physical system signals such as planetary gear fault signal and bearing life-cycle experiment signal. The ability of Von Neumann entropy and Rényi entropy for modal identification is studied in detail. Using the Von Neumann entropy and Rényi entropy, the coupling between two degrees of freedom in nonlinear physical system and degree of nonlinearity between fusion signals in different state systems are discussed. And the law between Rényi entropy and its scale parameter is also explored. In Section 5, we discuss the results. In Section 6, we summarize and suggest possible extensions to our work.

## 2. Theory

### 2.1. Von Neumann Entropy and Rényi Entropy

If there is entanglement between two microscopic particles in a common system, by measuring the properties of one of the particles, it is possible to predict the information of another particle that is entangled with the particle, thereby avoiding the interference of the measurement technique with the true physical state of the particle [66]. Entangled entropy reflects the degree of chaos of the entangled system and the uncertainty of the quantum system. Von Neumann entropy and Rényi entropy are a tool for measuring the degree of entanglement in the system. They can quantify the chaotic degree of the system and the nonlinear feature and coupling relationship between different systems. The algorithms for Von Neumann entropy and Rényi entropy applied to nonlinear mechanical systems are as follows.

Uncorrelated vectors are orthogonalized using Schmidt orthogonalization:

(1) $$\begin{array}{c} |\psi_{1}\rangle ={|I\rangle }_{1}\\ \vdots \\ |\psi_{k}\rangle ={|I\rangle }_{k}-\frac{\langle |\psi_{k}\rangle ,{|I\rangle }_{k-1}\rangle }{\langle {|I\rangle }_{k-1},{|I\rangle }_{k-1}\rangle }{|I\rangle }_{k-1} \end{array}$$

In the formula, ${\left\{I\right\}}_{k}$ is the right vector of vibration signal and ${\left\{\psi \right\}}_{k}$ is the orthogonal signal of vibration signal ${\left\{I\right\}}_{k}$.Then, the orthogonal vectors ${\left\{\psi \right\}}_{k}$ is transformed into the form of left vector and right vector.

The quantum state of the particle is described by the state vector function and exists in the Hilbert space in the form of complex vector. In a mixed state system, each subsystem appears with a certain probability, and there is no problem of coherence or interference between them. The entangled state cannot be written as the product state of all pure state subsystems, which is composed of multiple probability amplitude correlations in the non-fixed phase. The subsystems interact with each other and cannot be separated. In order to describe the state characteristics of quantum system conveniently, the density matrix is introduced. In the nonlinear mechanical system, the signal will inevitably be affected by many external factors, which aggravate the degree of nonlinearity and chaos. We hope to find a mathematical model to describe the state characteristics of nonlinear mechanical systems. Therefore, in this manuscript, the state density matrix [67,68] of the nonlinear mechanical system is established as follows. The Von Neumann entropy and Rényi entropy are used as quantitative indicators to quantify the degree of chaos, nonlinear characteristics and coupling, and analyzes the problems of nonlinear mechanical systems in modal identification, system time evolution and fault diagnosis:

(2) $$\rho =\sum_{k}|\psi_{k}\rangle p_{k}\langle \psi_{k}|$$

In the formula, $|\psi_{k}\rangle$ is a column vector, and $\langle \psi_{k}|$ is a transposed form of $|\psi_{k}\rangle$; in all directions, the probability of the sensor collecting the fault information is equal (i.e., $p_{k}=1/n$), and n is the number of subsystems.

In information theory, Renyi entropy is a function of τ, when τ takes different parameter values, it represents different types of entropy functions. That is to say, it includes Hartley entropy, Shannon entropy, collision entropy and minimum entropy. It is precisely that Renyi entropy has this generality, we should fully consider its limitations and scope of application. τ > 2 is a generalized correlation entropy, which has two limitations: All ranks of association refer to non-cross correlation and all ranks of association refer to non-memory association [69]. However, Renyi entropy and its included generalized entropy represent the uncertainty of information and measured the chaotic, nonlinear and coupled degree of the system.

Von Neumann entropy [67]:

(3) $$S_{V}=-tr(\rho \ln\rho )$$

Rényi entropy [68]:

(4) $$S_{\tau }(\rho )=\frac{1}{1-\tau }\ln tr(\rho^{\tau }),\;\;\tau \in Z_{+}\;\mathrm{and}\;\tau \neq 1$$

### 2.2. Compressive Sensing

#### 2.2.1. Theory

Compressed sensing exploits the sparseness and compressibility of a signal in a certain domain. The original signal is observed with an observation matrix that is uncorrelated with the transform base to form an observation value in a low-dimensional space. Finally, the reconstruction signal is obtained by solving the convex optimization problem with known measurement matrix, transformation basis and observation value. Improving the independent randomness between each observation base and the degree of incoherence, between the observation base and the signal, the measurement number and length of the observation value will be optimized accordingly, making the reconstruction result more accurate. This manuscript will use the noise reduction capability [70] of compressed sensing to reconstruct the acquired signal to reduce the noise impact.

Compressed sensing is divided into three main steps [53]:

(1) Sparse representation of the signal.
(2) The observation matrix is designed to ensure that the dimension is reduced and the loss of signal characteristics is minimized.
(3) By using the minimum L_0 norm optimization algorithm, the approximate sparse coefficient is obtained, and the *X* is restored from the observed value *y*.

Let one-dimensional discrete N×1 time signal X:

(5) $$X={\left(x_{1},x_{2}......x_{N}\right)}^{T}$$

The signal X is sparse by using the sparse basis matrix Ψ:

(6) $$\Psi =\left[\psi_{1},\psi_{2}\ldots \psi_{N}\right]$$

(7) $$s_{i}=\langle X,\psi_{i}\rangle =\psi_{i}{}^{T}X$$

(8) $$X=\sum_{i=1}^{N}s_{i}\psi_{i}=\Psi s$$

where $\psi_{i}$ is the vector of N×1 orthogonal to each other; $s_{i}$ is the sparse coefficient of N×1 and K,$s_{i}$ are non-zero values.

The original signal {x} is observed by using the observation basis ${\left(\phi_{1},\phi_{2}\cdots \phi_{M}\right)}^{T}$ of the random observation matrix Φ. The random observation matrix Φ should not be related to the sparse basis matrix and the low dimensional observation value M×1 of G is obtained:

(9) G=〈{x},Φ〉

(10) G=Φ{x}=ΦΨs=Θs

where Θ is the sensing matrix.

Limited to equal features:

(11) $$1-\varepsilon \leq \frac{{\left\|\Theta u\right\|}_{2}}{{\left\|u\right\|}_{2}}\leq 1+\varepsilon$$

where, some ε > 0.

The $l_{0}$ norm ${\left\|s^{\prime }\right\|}_{0}$ of vector $s^{\prime }$ represents the number of non-zero elements in $s^{\prime }$. Because the more sparse the sparse coefficient is, the more accurate the reconstructed signal is. Among the many $s^{\prime }$ satisfying $\Theta s^{\prime }=G$ condition, $s^{\prime }$ with minimum $l_{0}$ norm is the optimal sparse coefficient needed for reconstruction:

(12) $$\hat{s}=\arg\min{\left\|s^{\prime }\right\|}_{0}\;\;s.t\;\Theta s^{\prime }=G$$

$s^{\prime }$ is the estimated value of sparse coefficient s, then the reconstructed signal is $\left\{x^{\prime }\right\}=\psi s^{\prime }$.

#### 2.2.2. Analysis of Noise Reduction Performance

In order to verify the sparse property and noise reduction performance of the compressed sensing technology, a simulation signal *f*(*t*) composed of trigonometric function, exponential modulation sine function, *sinc* function and *diric* function is constructed:

*f*(*t*) = 0.03*e*^0.5*t*^*cos*(10*πt* − 1) + 4.78*sinc*(*t*) + 5*diric*(*t*) + 2.5*sin*(2*πt*) + 3.11*cos*(20*πt*) (13)

**Figure 1:**
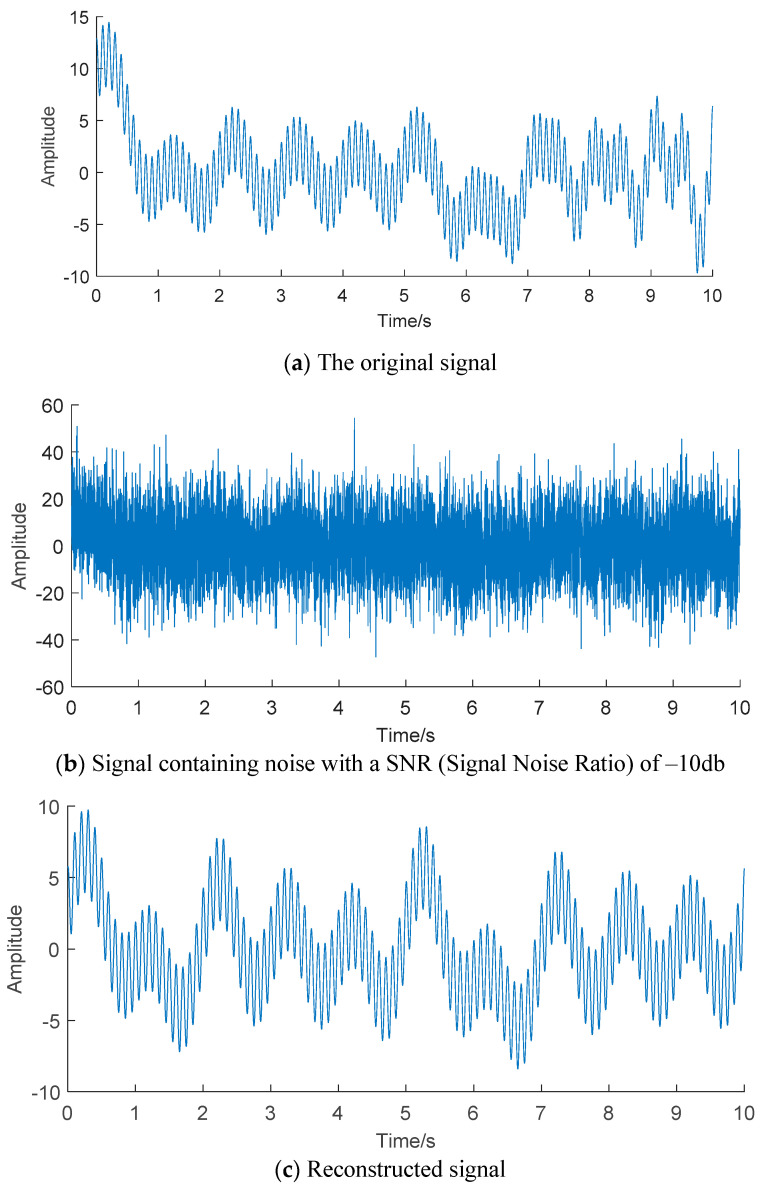
Processing results of simulated signal by compressed sensing technique.

The simulation signal is shown in Figure 1a. Add Gaussian white noise with signal-to-noise ratio of −10 dB to the simulation signal, and the time domain diagram with noise is shown in Figure 1b. The noise reduction process is performed on the simulation signal by using the compressed sensing technology, and the obtained result is shown in Figure 1c. The figure shows that the reconstructed signal basically guarantees the synchronization of the original signal feature elements. The Pearson correlation coefficient between the original signal and the reconstructed signal is 0.8927. At the time of 0.301 s, 1.2 s, 2.207 s, 3.302 s, 4.202 s and 5.303 s, the amplitudes of reconstructed signals are 9.726, 3.025, 7.635, 5.637, 4.6 and 8.545, respectively. At the time of 0.2 s, 1.202 s, 2.2 s, 3.305 s, 4.2 s and 5.203 s, the amplitudes of the original simulation signals are 14.31, 3.616, 6.272, 5.159, 4.969 and 6.235 respectively. The results show that the compressed sensing technology based on signal sparsity and non-correlation can effectively reconstruct the original signal with high accuracy from redundant and complex strong noise, and has strong robustness to noise.

The flow of the method proposed in this paper is as follows:

(1) The acceleration signal of the mechanical system is collected by the acceleration sensor: the *x*-direction signal and the *y*-direction signal.
(2) The compressed sensing technology is used to reconstruct and reduce the noise of the signal.
(3) The state density matrix of the nonlinear mechanical system is constructed, and the degree of coupling between two degrees of freedom signals is calculated by using Von Neumann entropy and Rényi entropy, respectively.

**Figure 2:**
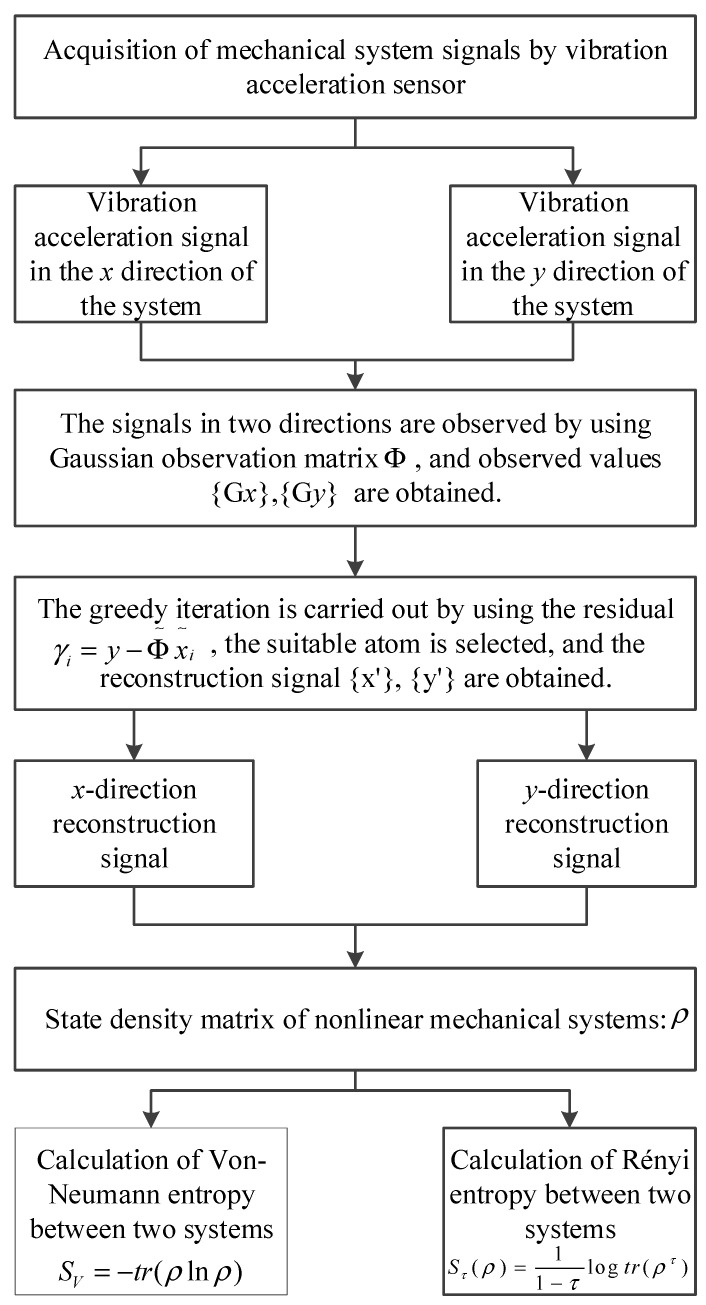
The flowchart of the method proposed in this paper.

The flowchart of the method presented in this paper is shown in Figure 2.

## 3. Chaotic System Analysis

Chaotic system refers to the existence of random irregular motion in a deterministic system, and its behavior has three characteristics: uncertainty, non-reproducibility and unpredictability. In 2002, Lü and Chen discovered the Lü chaotic system through the idea of chaotic anti-control, which established a bridge between the Lorenz system and the Chen system [71,72]. The dynamic equation of the Lü chaotic system is:

(14) $$\begin{array}{l} \dot{x}=a(x-y)\\ \dot{y}=-xz+cy\\ \dot{z}=xy-bz \end{array}$$

**Figure 3:**
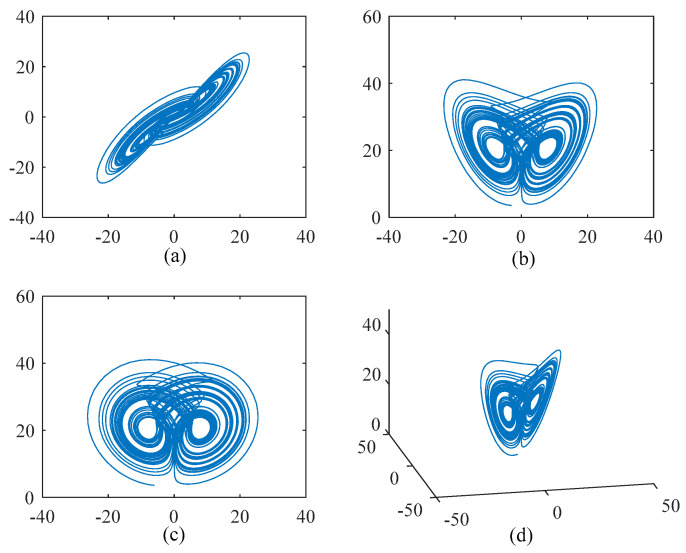
Phase diagram and three-dimensional diagram of attractor of Lü chaotic system. (**a**) *x*–*y* phase plane strange attractors. (**b**) *x*–*z* phase plane strange attractors. (**c**) *y*–*z* phase plane strange attractors. (**d**) Three-dimensional view.

Similar to the Lorenz system and the Chen system, the Lü system enters a chaotic state when the parameters select certain values. *a*, *b* and *c* are the three control parameters of the Lü chaotic system. When the parameters *a* = 36, *b* = 3, *c* = 20, the dynamic behavior of the system presents a chaotic state [72]. We give *x*, *y*, and z initial values of −3, −6, and 3.6, respectively, and the Lü chaotic system attractors are shown in Figure 3. The phase diagram and three-dimensional image of the chaotic attractor are shown in Figure 3.

In the Lü chaotic system, the parameter *c* plays a controlling role in the whole system. The dynamic behavior of the Lü chaotic system has obvious stage with the change of the control parameter *c*. When 12.7 < *c* < 17.0, the attractor generated by the system similar to the Lorenz chaotic system attractor; it has a transient shape when 18.0 < *c* < 22.0; and becomes similar to the Chen chaotic system attractor [73,74] when 23.0 < *c* < 28.5.

**Figure 4:**
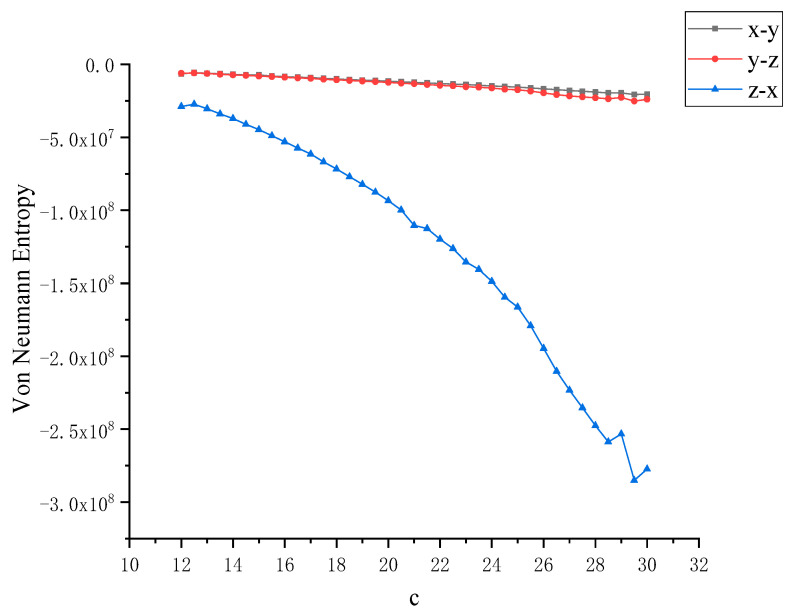
Von Neumann entropy between *x*, *y*, *z* with the variation of control parameter *c* of Lü chaotic system.

**Figure 5:**
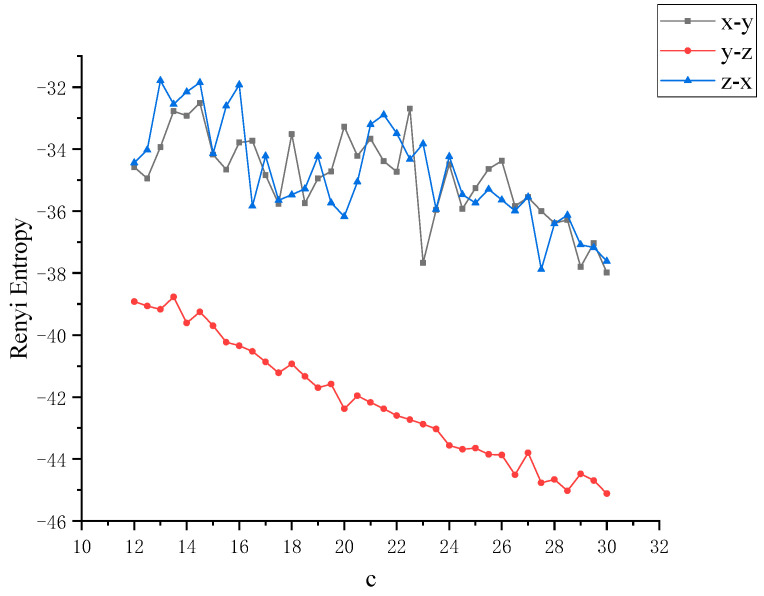
Rényi entropy between *x*, *y*, *z* with the variation of control parameter *c* of Lü chaotic system.

**Figure 6:**
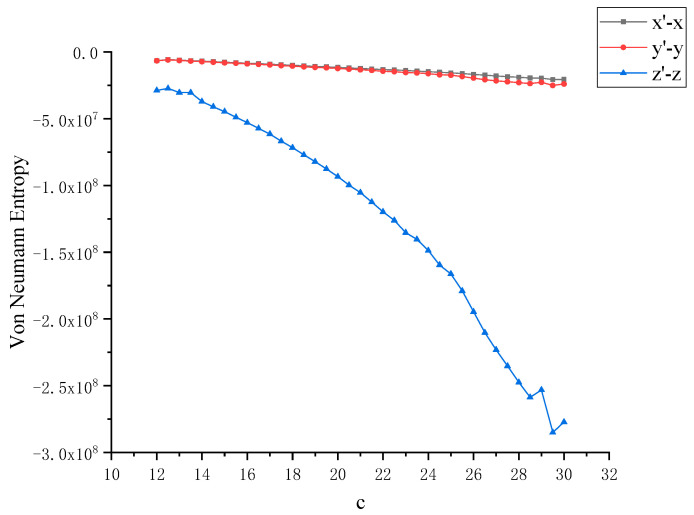
Von Neumann entropy between *x*, *y* with the variation of chaotic system control parameter *c* and *x*, *y* at *c =* 12 (that is, non-chaotic state).

**Figure 7:**
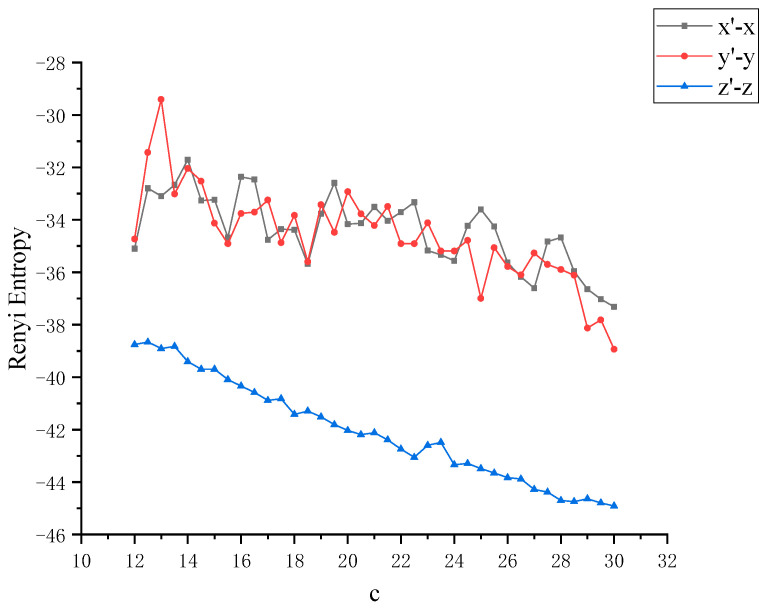
Rényi entropy between *x*, *y* with the variation of chaotic system control parameter *c* and *x*, *y* at *c* = 12 (that is, non-chaotic state).

In order to further study the chaotic characteristics of the Lü system, in the simulation of dynamics, the control parameter *c* is changed between [12,30] with a step size of 0.1. Considering that Von Neumann entropy and Rényi entropy are good dimensionless measures, we will dynamically compare the two metrics and calculate the Von Neumann entropy and Rényi entropy between *x*, *y* and *z* as the control parameter *c* changes, as shown in Figure 4 and Figure 5. It can be observed that this nonlinear system shows complex and rich chaotic dynamics behavior. When the parameter *c* of the chaotic system changes, the coupling degree between *x*, *y* and *z* also changes. In this process, the non-chaotic and chaotic states of the system are included. But you can also see that all entropy values are negative, and negative entropy indirectly illustrates the degree of chaos in the system through a new angle. For chaotic systems, the negative entropy drawn from the external system cannot reduce its own entropy increase like life body to maintain the orderly development of the system. Negative entropy can be explained as the amount of definite information needed for the transformation of chaotic systems to non-chaotic systems. The larger the amount of information required by the system means that the more uncertain factors in the chaotic system, the more chaotic the system. When *c* = 12, the Lü chaotic system does not enter the chaotic state, and the coupling degree of *x*-*y* and *y*-*z* is large, and Von Neumann entropy value fluctuates near 0. With the increase of *c*, the system enters the chaotic state, and the coupling degree between *x*, *y* and *z* decreases rapidly, and the value is further away from 0, because they are further away from zero in the longer time evolution. This is because nonlinear interactions and more states contribute greatly to the evolution of Von Neumann entropy and Rényi entropy. Next, the coupling degree between *x*, *y*, *z* in Lü chaotic system is further reflected by calculating two kinds of entropy. The range of parameter *c* varies from [12,30]. We discuss that the Von Neumann entropy and Rényi entropy of Lü system with the variation of chaotic system control parameter *c* and *c* = 12 (that is, non-chaotic state) are shown in Figure 6 and Figure 7, which are similar to those in Figure 4 and Figure 5.

**Figure 8:**
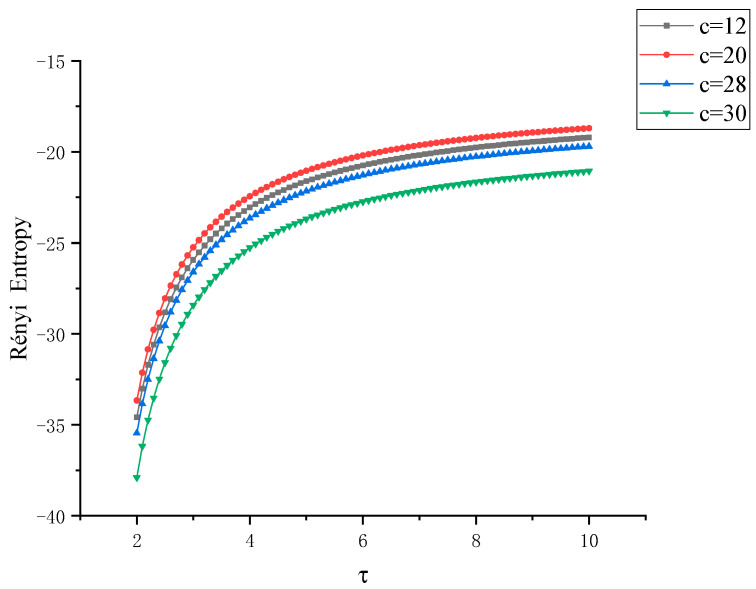
Curve of Rényi entropy between *x*-*y* with control factor τ when the control parameter *c* of Lü chaotic system takes different eigenvalues.

**Figure 9:**
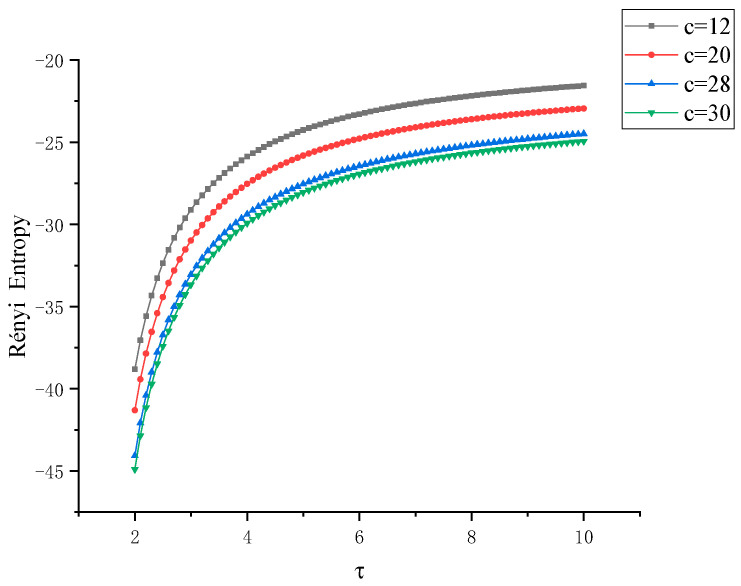
Curve of Rényi entropy between *x*-*z* with control factor τ when the control parameter *c* of Lü chaotic system takes different eigenvalues.

Next, from another perspective, we use the Rényi entropy at different scales to study the Lü chaotic system in a comprehensive and three-dimensional way. When the characteristic parameter *c* is taken as 12, 20, 28, 30, respectively, the Rényi entropy images of *x*-*y* and *y*-*z* are obtained, as shown in Figure 8 and Figure 9, respectively, wherein the parameter τ of the Rényi entropy are in a changing state. In the four states, the Rényi entropy increases rapidly in the interval τ∈ [2, 5] with the increase of the scale parameter τ. In the interval τ∈ [5, 10], the growth rate of Rényi entropy of the four states slows down and converges to a value nearby. Generally, it shows the trend of Rényi entropy*_c=_*_12_ > Rényi entropy*_c=_*_20_ > Rényi entropy*_c=_*_28_ > Rényi entropy*_c=_*_30_. The two kinds of entropy measures can accurately distinguish several different states of Lü system and the coupling degree among *x*, *y*, *z*, which is the three degrees of freedom data of the system.

The coupling degree measures of Von Neumann entropy and Rényi entropy can play an important and roughly the same role in the study of the chaotic characteristics of the Lü system. These two measurement methods can provide coupling measurement for a standard nonlinear system and accurately judge the state of the chaotic system. Von Neumann entropy and full-vector multi-scale Rényi entropy are robust enough to the change of Lü chaotic system state. However, in the signal processing of nonlinear mechanical system, more research is needed on Von Neumann entropy and Rényi entropy.

## 4. Experimental Study

When the key transmission components of large mechanical equipment, such as rolling bearings and planetary gears, fail, the vibration signal has typical strong nonlinear characteristics. Next, the rotational mechanical nonlinear system is used to verify the validity, superiority and universality of Von Neumann entropy and Rényi entropy in nonlinear mechanical systems. In order to further study the coupling behavior of signals in nonlinear mechanical systems, the following two rotating machinery experiments are used: (1) Accelerated life test of rolling bearing and (2) Fault Diagnosis Experiments of Planetary Gear Transmission System. The above two experiments have typical strong nonlinear characteristics, which are very suitable for studying the application of Von Neumann entropy and Rényi entropy in real mechanical systems.

### 4.1. Accelerated Life Test of Rolling bearings

#### 4.1.1. Introduction to the Experiment

The IEEE Reliability Association and the Femto-st Institute of France organized the IEEE PHM rotating Machinery Fault Prediction Challenge in 2012. The challenge dataset was provided by Femto-st Institute and tested on PRONOSTIA’s bearing accelerated aging platform [75].

**Figure 10:**
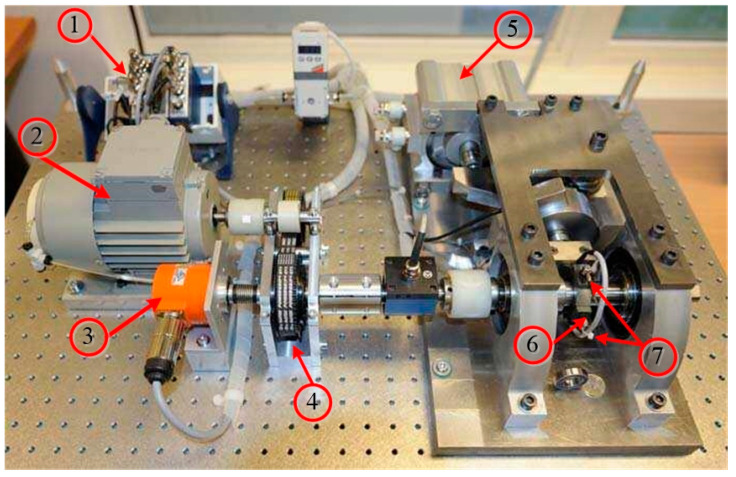
PRONOSTIA bearing accelerated Aging Test bench. (1: Acquisition system; 2: AC motor 3: Speed recorder; 4: Retarder; 5: Pneumatic jack; 6: Test bearing; 7: Accelerometers).

The “PRONOSTIA” test bench simulates the natural degradation process of the bearing during its service life [76]. The structure of the test bench is shown in Figure 10. The main goal of the experiment was to provide real experimental data to characterize the degradation of the ball bearing over its lifetime [76]. The bearing type to be tested is NSK 6804RS, and two acceleration sensors are arranged on the outer ring of the bearing to synchronously collect the horizontal and vertical vibration signals of the experimental platform. The operating conditions of the test bench are as follows: The motor speed is 1800 rpm. The sampling frequency is 25.6 kHz sampling, and a radial force of 4000N is applied to the rotating shaft and the bearing to be tested [77].

#### 4.1.2. Data Analysis

In the above, we have done a detailed study on the coupling relationship and chaotic evolution in chaotic systems. Next, in the evolution of the dynamics state of the mechanical system, we separately study the coupling relationship between the two vertical degrees of freedom signals in the same system and the coupling relationship between the full vector signals of mechanical systems in different states.

**Figure 11:**
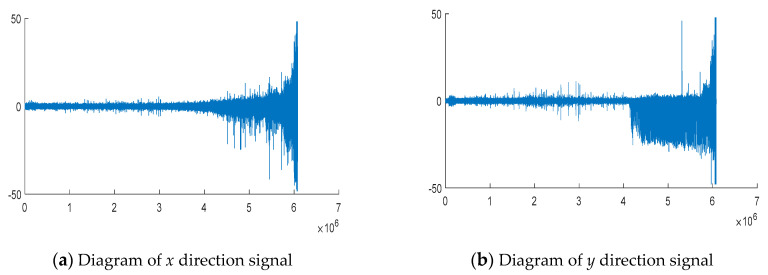
The raw data collected from PRONOSTIA bearing accelerated aging test.

Figure 11 shows the raw vibration signals in two orthogonal directions collected from PRONOSTIA throughout the experiment. 

**Figure 12:**
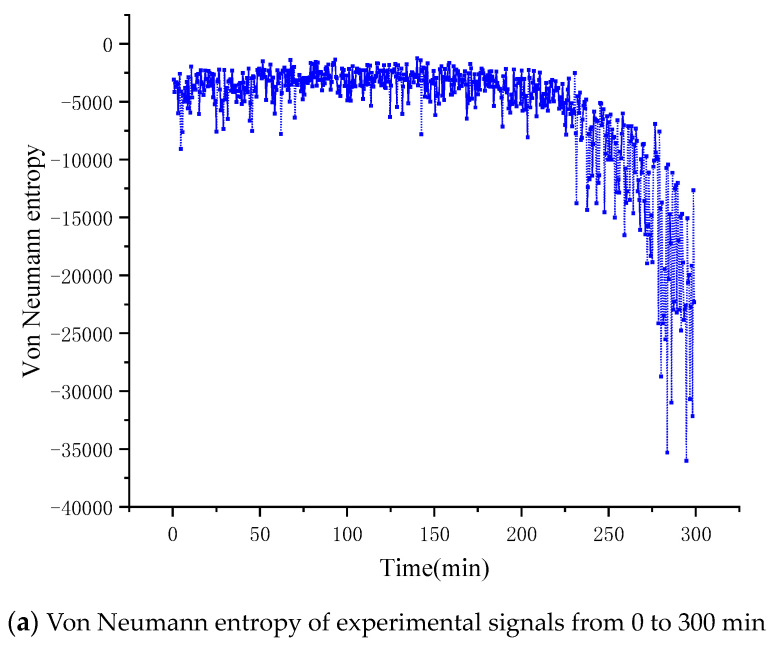

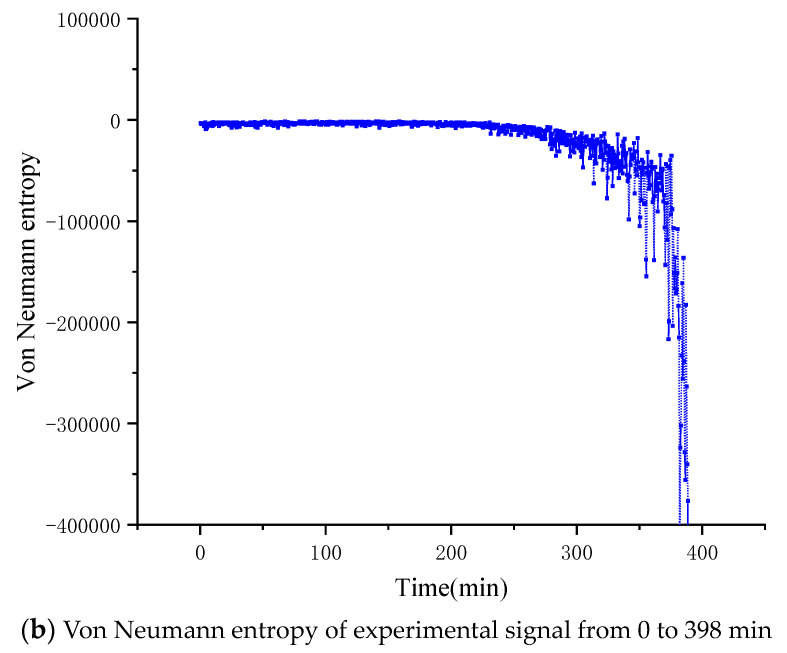
Von Neumann entropy of experimental signal.

**Figure 13:**
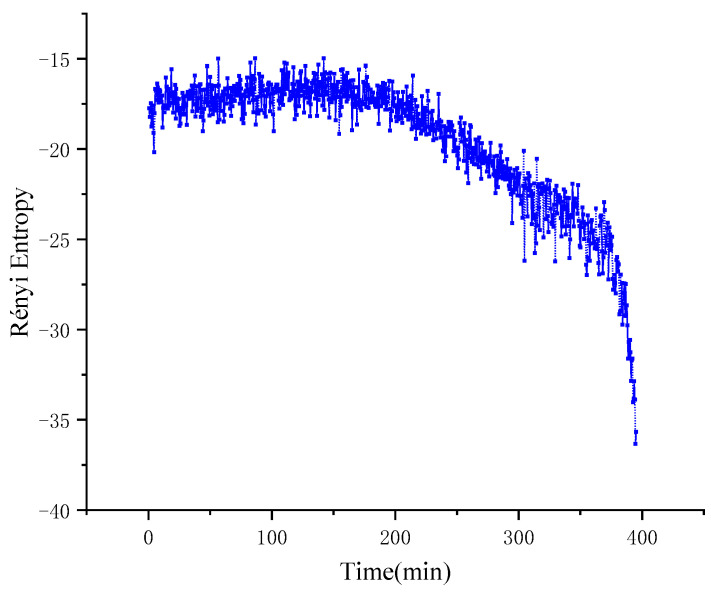
Rényi entropy of experimental signal.

**Figure 14:**
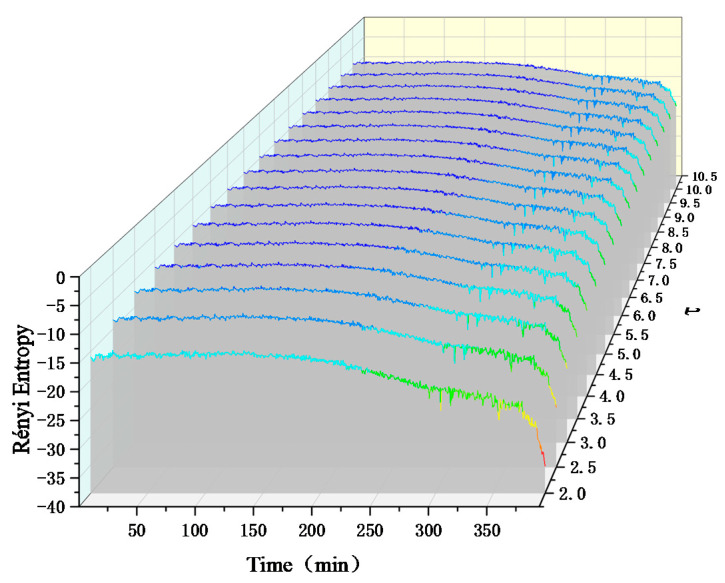
Rényi entropy of experimental signals at different scales.

The vibration signals of the accelerated life test of the bearing in the *x* and *y* directions are shown in Figure 11a,b, respectively. Next, we will discuss the signal coupling degree of the mechanical system in *x*, *y* degrees of freedom, and then determine the operating state of the mechanical system through inversion. Figure 12 and Figure 13 show the entropy calculated by Von Neumann entropy and Rényi entropy with scale parameter 2, respectively. In Figure 14, in 0–230 min, the Von Neumann entropy between the *x*-direction and *y*-direction vibration signals is stable at about −0.5 × 10^−4^. Compared with the later entropy development trend, it can be said that the Von Neumann entropy before the 230th minute is stable in a relatively high range, and the coupling degree of the two degrees of freedom signals is relatively high, which reflects that the mechanical system is currently in a relatively disordered state, further explaining the health of the mechanical system. After that, the entropy decreases rapidly. In 230–400 min, the Von Neumann entropy decreases from −0.5 × 10^−4^ to −3.5 × 10^−4^, the coupling degree between two degrees of freedom signals changes obviously, and the nonlinear characteristics of the mechanical system are enhanced, which shows the evolution process of the occurrence and development of mechanical system faults.

In Figure 13, in the 0–200 min region, the Rényi entropy between the two degrees of freedom vibration signals is stable at about −18. At this time, the operation state of the mechanical system is stable, and the coupling degree between the signals on the two degrees of freedom is high. Compared with Von Neumann entropy, Rényi entropy discovered the state mutation of mechanical system about 30 min earlier. Within 200 to 300 min, the Rényi entropy roughly decreases linearly to −25; in 300–375 min, the rate of decline of entropy slows down and drops sharply after the 375th minute. From 200 to 300 min, the entropy of Rényi decreased to about −25 in a linear trend roughly; from 300 to 375 min, the entropy decreased slowly, and then decreased rapidly after 375 min. The trend of Rényi entropy first reflects the change of coupling degree of the signals on the two degrees of freedom of *x* and *y*, which reflects the evolution process of the degree of nonlinearity in the mechanical system from small to large.

We observe that the two measure entropies are consistent in judging the evolution trend of bearing faults, but compared with Von Neumann entropy, Rényi entropy can better show the change process of bearing mechanical state, and the effect is more accurate. In the case of Rényi entropy of different scales, the coupling relationship between bearing signals is shown in Figure 14. The occurrence time of faults is about 200 min and the trend is similar. However, as the entropy scale of Rényi increases, the amplitude change of entropy will gradually decrease, which is not conducive to the observation and judgment of macroscopic physical nonlinear systems.

In Figure 12, Figure 13 and Figure 14, Von Neumann entropy and Rényi entropy can invert the dynamic changes of the system on the basis of the order degree of information. When the bearing fails, the bearing will produce periodic signal with characteristic frequency, and the degree of nonlinearity is high, and with the evolution of fault degree, the proportion of characteristic signal in the system will gradually increase. Compared with the mechanical system in a healthy state, the regularity of the internal signal of the system will be significantly enhanced and the value of entropy will significantly decrease. Von Neumann entropy and Rényi entropy is a tool to measure the order degree of the system. The more ordered the system is, the smaller the value of entropy is. This also explains the phenomenon that the higher the degree of nonlinearity of the mechanical system is, the smaller the value of entropy is.

In the macroscopical world, due to the interference and modulation of external factors, the system signals tend to be nonlinear, which brings more challenges to the attribute recognition and prediction of physical macroscopic nonlinear systems. Figure 12 and Figure 13 present the dynamics coupling between the *x* and *y* two degrees of freedom signals in the bearing drive train. By studying the occurrence time of bearing failure and the evolution process of bearing dynamics, we verify the ability of the macroscopic physical nonlinear system to judge the attributes of the macroscopic system through the coupling relation generated by the dynamic signals with different degrees of freedom in itself. We have studied the variation of the coupling degree between the *x* and *y* two-degree-of-freedom signals of the mechanical system during the evolution of the dynamics state, and analyzed the rules and connotations in detail. Next, we will study coupling degree between different states of the nonlinear system through the experiment of “planetary gearbox fault diagnosis test bench”.

### 4.2. Fault Diagnosis Experiment of Planetary Gear Transmission System

#### 4.2.1. Experimental Introduction

Studying more complex mechanical systems is a challenging task. Compared with other mechanical systems, the gear transmission system of planetary gear has some unique characteristics, such as high signal complexity and high degree of non-linearity. The fault diagnosis test-bed of planetary gearboxes can deeply study a complete nonlinear power transmission system [78].

**Figure 15:**
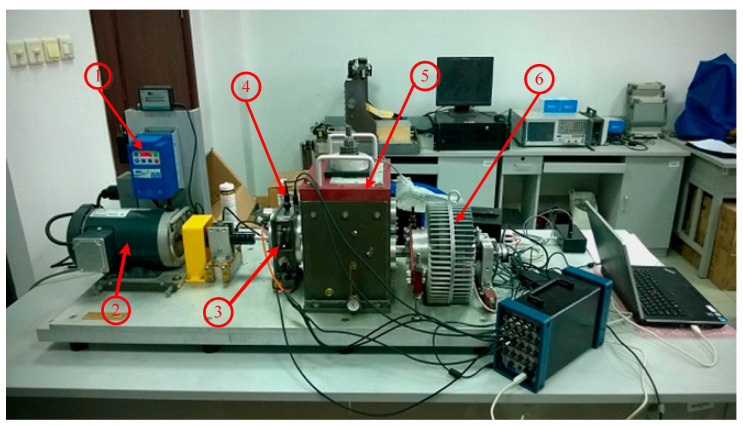
Fault diagnosis test bench for planetary gear boxes. (1: Controller; 2: AC motor 3: Planetary gearbox; 4: Accelerometers; 5: Gearbox; 6: Magnetic powder brake).

As shown in Figure 15, the test bench includes a two-stage planetary gearbox, a parallel shaft gear box, a bearing load and a programmable magnetic excitation brake. The test bench can simulate the gear tooth breaking, tooth surface wear, gear tooth crack, tooth surface pitting corrosion and tooth missing of the gear [79,80,81]. The vibration acceleration signals of five kinds of planetary gears in different states are collected [82].

#### 4.2.2. Data Analysis

Whether the Von Neumann entropy and Rényi entropy calculated by mechanical dynamics system will change in physical meaning or not when the Rényi entropy is at different scales, and what laws will appear? Whether the idea of modal recognition is still vaild by calculating the Von Neumann entropy and Rényi entropy between mechanical dynamics signals. Therefore, we will analyze the coupling degree between the fusion signals in the planetary gearboxes of five different states, and examine and explore the above problems.

**Figure 16:**
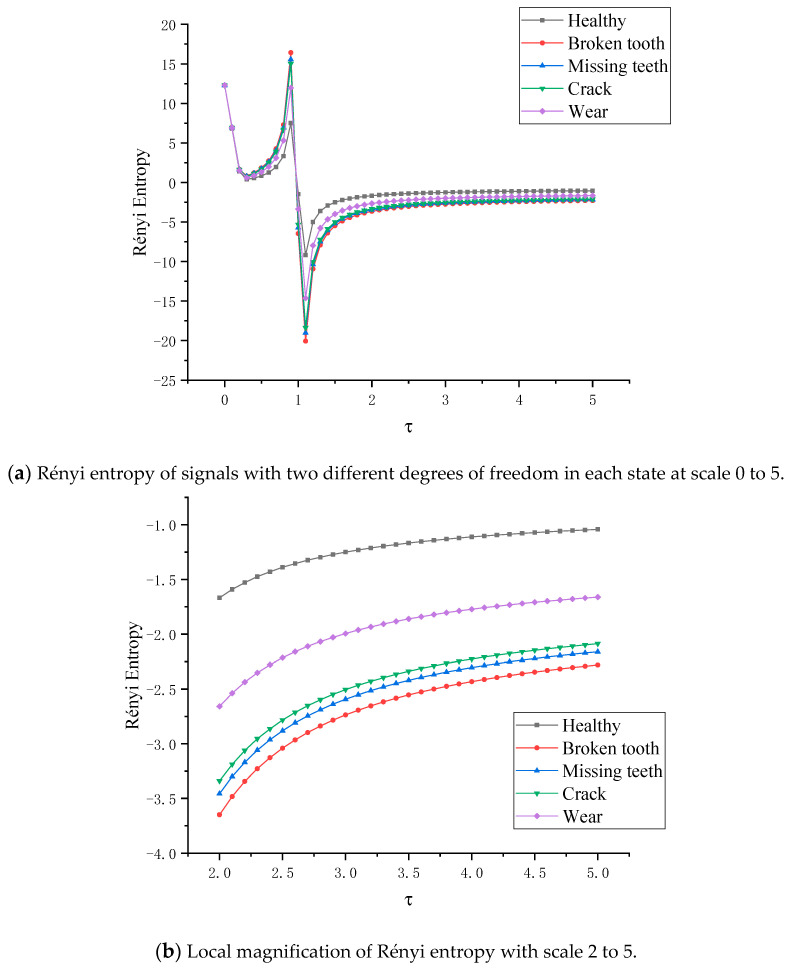
Rényi entropy of two different degrees of freedom signals in each state at scale 0 to 5.

When the planetary gear transmission system is in five different states, the Rényi entropy of the two vertical degrees of freedom signals in each state is shown in Figure 16a, where the scale of the Rényi entropy is τ∈ [0, 5]. As shown in the Figure 16a, when τ∈ [0, 0.3], the entropy is decreasing and greater than zero, and the entropy of the planetary gears in different states is almost as difficult to distinguish in this scale. When τ∈ [0.3, 0.9], the entropy is positive and positively correlated with the scale parameters and reaches the peak value at τ = 0.9. The five states of planetary gears are distinguished. The peak values are about 7.5037, 11.963, 15.0302, 15.555 and 16.417, respectively. When τ∈[0.9,1), the entropy decreases sharply, completes the transformation from positive entropy to negative entropy, and reaches the minimum at τ = 1.1. The state of planetary gears is distinguished, and the peaks are about −9.171, −14.621, −18.37, −19.012, −20.0654, respectively. When the Rényi entropy scale τ∈ [0, 5], the entropy value of each state increases and keeps the fault order. When τ = 5, the entropy of each state converges to a certain value, which is about −1.04, −1.60, −2.05, −2.15, −2.25. In order to judge the fault order more clearly, the scale parameter of Rényi entropy is taken to [2, 5] for local amplification, as shown in Figure 16b. The negative entropy represents the energy that the gear absorbs from the outside world in order to resist the increase of its own entropy, and indirectly represents the chaos degree of the system state. On any parameter scale, the fault degree order of the five states remains unchanged, just the physical meaning of positive entropy and negative entropy is different.

In the accelerated life experiment of the rolling bearing and the fault diagnosis experiment of the planetary gear, we prove that the physical characteristics of the nonlinear mechanical system can be analyzed by the coupling effect between the signals of different degrees of freedom under a single working condition. However, Von Neumann entropy and Rényi entropy are still used as the measurement indexes of the physical characteristics of nonlinear mechanical systems. Whether the state characteristics of nonlinear mechanical systems can still be determined through the coupling effects between nonlinear mechanical systems in different states? Can Rényi entropy still perform modal recognition processing on nonlinear mechanical systems when scale parameter τ changes? When the scale parameter τ changes, what is the law of Rényi entropy? Based on this, we construct a full-vector multi-scale Rényi entropy.

The feature parameters extracted by homologous information fusion meet the data calibration in time and space. When the sampling frequency is high enough, full vector spectrum technology can overcome the shortage of information caused by short-time Fourier transform. For the feature information extraction of rotating rigid body motion state of mechanical system, the single channel information from single sensor cannot describe its state feature comprehensively and accurately, but through the multi-channel data from multiple sensors, it can reflect the motion information of rotor from multiple directions, make up for the information defects and eliminate the uncertain factors, so the fusion of homologous information is a key technology. Based on the same source signal, the full vector spectrum technology shows the harmonics and their amplitudes in the form of spectrum chart. In the case of ensuring the high resolution of the spectrum analysis, the fault characteristics of the mechanical system are intuitively reflected [83].

The process of full vector Rényi entropy is as follows:

(1) Acceleration signals in different states of the same system are collected and obtained by accelerometer: two signals *x*_1_, *y*_1_ in normal state, *x*_2_ and *y*_2_ in relative fault state.
(2) The compressed sensing technology is used to reconstruct and reduce the noise of the signal.
(3) Vector fusion of homologous signals
(4) Construct state density matrix of nonlinear mechanical system and calculate the Rényi entropy between the two systems at different scales.

**Figure 17:**
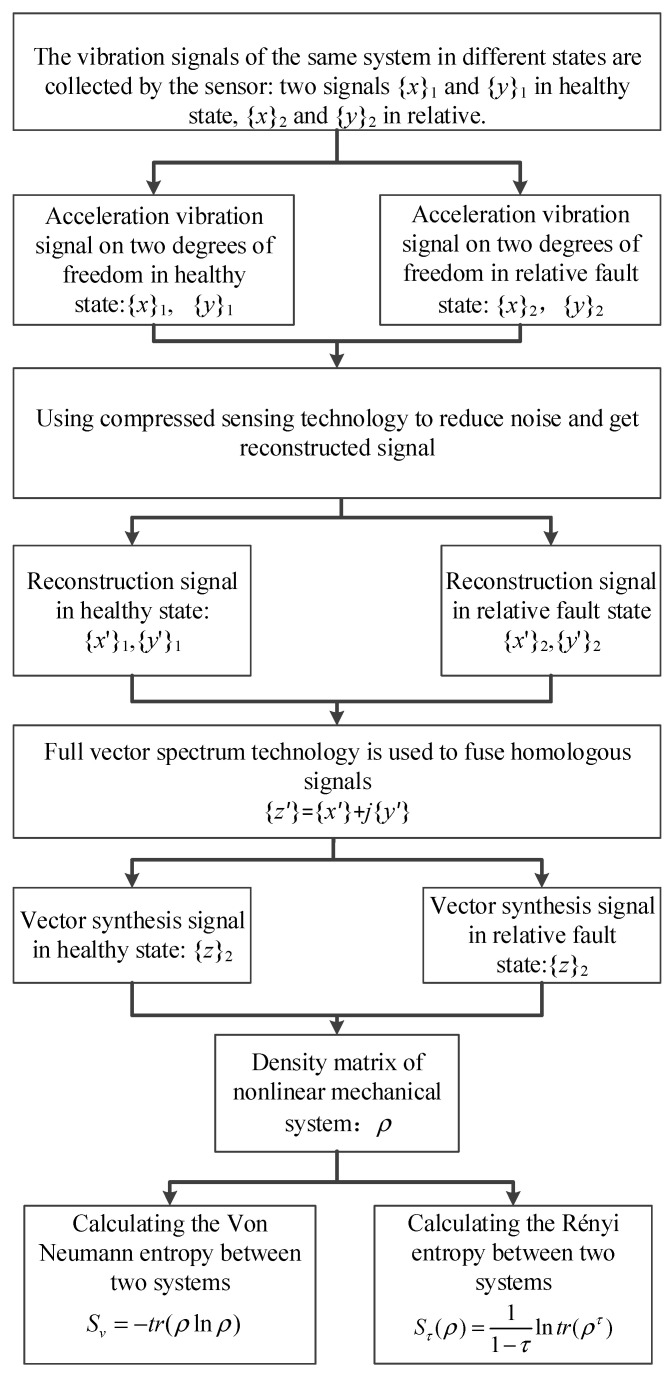
Full-vector Rényi entropy flowchart.

The full vector Rényi entropy flowchart is shown in Figure 17.

**Figure 18:**
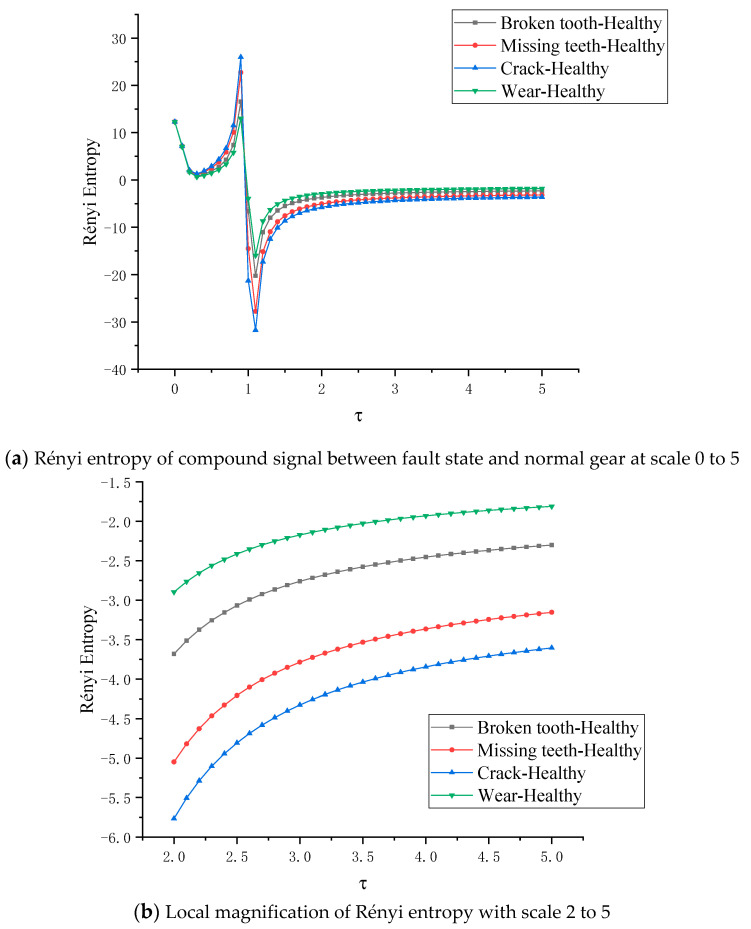
Rényi entropy of compound signal between fault state and normal gear at scale 0 to 5.

The density matrix between each fault state planetary gear and the healthy state planetary gear is constructed and the full vector multi-scale Rényi entropy is calculated. Figure 18a shows the full vector multiscale Rényi entropy image of wear-healthy, broken tooth-healthy, missing tooth-healthy and root crack-healthy respectively, where the scale parameter of full vector Rényi entropy is [0–5]. When τ∈[0,0.3], the entropy value decreases and is greater than zero. In this parameter interval, the value of Rényi entropy between fusion signals in different planetary gearboxes is almost the same When τ∈[0.3,0.9], the entropy value is greater than zero and positively related to the scale parameter and reaches the peak value at τ=0.9. Four kinds of planetary gear fault states are distinguished. The peaks of the systems of wear-healthy, broken teeth-healthy, missing teeth-healthy and crack-healthy are about 0.654, 0.822, 1.115 and 1.2695, respectively.

When τ∈[0.9,1), the entropy drops sharply, completing the transition from positive entropy to negative entropy, and reaching the minimum value at τ=1.1. The peak value of Rényi entropy of fusion signal in planetary gearbox under four fault states is about −15.930, −20.247, −27.76, −31.721, respectively. When τ∈(1,1.5], the entropy value of each state increases and keeps the original fault order. When τ→5, the entropy values of the fused signals in the planetary gearboxes of each state converge to a fixed value, which is approximately −1.80, −2.3, −3.1, −3.5. In order to judge the fault order more clearly, the scale parameter of the Rényi entropy is taken to τ∈[2,5], as shown in Figure 18b. It can be seen that the state features of the nonlinear mechanical system can still be quantified by the coupling effect between nonlinear mechanical systems in different states; the change of the scale parameter τ does not affect the modal identification of the nonlinear mechanical system; The Rényi entropy changes with the change of the scale parameter τ and presents a certain law.

## 5. Discussion

The dynamic evolution processes of Lü chaotic systems and two typical mechanical systems are studied in this manuscript. The dynamics behaviors of these two kinds of nonlinear systems are characterized by uncertainty, repeatability and unpredictability. At the same time, these two kinds of nonlinear systems have certain similarities. For example, when the Lü chaotic system is in chaotic state, the coupling degree between subsystems decreases rapidly, and the dynamic behavior is highly nonlinear. When the mechanical system is in the fault state, the regularity of the information in the system is enhanced and the coupling degree decreases. The characteristic signals of the two kinds of systems are highly nonlinear and unstable. Therefore, in the manuscript, we discussed in detail the coupling of Lü chaotic system, the change of coupling degree between two degrees of freedom signals of mechanical system in the process of dynamics state evolution, and the coupling relationship between different states of planetary gear transmission system.

In the Lü chaotic system, we take the Von Neumann entropy and Rényi entropy as measures to study the complex and rich chaotic dynamic behavior of the nonlinear system. Von Neumann entropy and Rényi entropy is used to analyze the chaotic characteristics and coupling strength between the causal information of Lü’s chaotic system, and to compare their dynamics behaviors in different initial states. When exploring the change of coupling degree between two degrees of freedom signals in the evolution of dynamics state of mechanical system, we measured the same mechanical system with Von Neumann entropy and Rényi entropy respectively, and find that both of them can respond well to the change of nonlinear degree of the system, but Rényi entropy works better. Then we measure the bearing transmission system with Rényi entropy of different scales and find that with the increase of scales, the dynamics evolution process is more gentle, which is not conducive to the pick-up of dynamics characteristics.

When studying coupling relationship between the different states of planetary gear transmission system, we use Rényi entropy of different scales as a measure tool. We find that when τ∈[0.9,1), the entropy changes from positive entropy to negative entropy, and with the increase of Rényi entropy scale, the Rényi entropy of systems in different states will approach a fixed value infinitely. Both positive entropy and negative entropy explain the degree of nonlinearity in planetary gear transmission systems in different states. Negative entropy represents the energy that individuals draw from the outside world in order to reduce their own entropy increase. In the absence of external intervention, the entropy owned by the individual will increase, and when the entropy reaches a certain value, the individual will die out. When the negative entropy of the nonlinear mechanical system increases, which shows that the system is developing towards strong nonlinearity. In the course of experimental exploration, we have found some interesting properties of Rényi entropy: With the change of Rényi entropy scale parameters, the positive and negative value of entropy may change, but this does not affect the judgment of the dynamics behavior of nonlinear mechanical dynamics systems. With the increase of the scale parameter of Rényi entropy, the Rényi entropy of the same state system converges to one fixed value. 

Von Neumann entropy and Rényi entropy can accurately measure the degree of disorder of nonlinear systems, and provide important information for analyzing the dynamic properties of nonlinear mechanical dynamic systems. Von Neumann entropy and Rényi entropy can analyze the characteristics of strong nonlinearity, instability and strong coupling of signals, extract sensitive characteristic variables of signals, perform modal identification on nonlinear mechanical dynamic systems, and trace and describe the evolutionary state of mechanical dynamic systems.

## 6. Conclusions

In this manuscript we propose a new method to describe the nonlinear features and coupling relationship of mechanical systems, and establish a state density matrix of nonlinear mechanical systems, using Von Neumann entropy and Rényi entropy as measurement indicators to quantify the degree of chaos, nonlinear characteristics and coupling between nonlinear systems. We apply the proposed method to Lü chaotic systems and two typical nonlinear mechanical systems, and study the capabilities of Von Neumann entropy and Rényi entropy in pattern recognition. For the more complex planetary gear transmission system, we extend the coupling system on this basis, and propose a new nonlinear system state measurement index-full vector multi-scale Rényi entropy, and discuss the degree of nonlinearity and the coupling relationship among several nonlinear mechanical systems. The research results build a bridge between nonlinear mechanical system dynamics and information dynamics, and prove the effectiveness of the proposed method in modal identification, system dynamics evolution and fault diagnosis of nonlinear systems. The conclusions are as follows:

(1) The state density matrix we have established can well describe the state features of nonlinear mechanical systems in practical tests. The Von Neumann entropy and Rényi entropy are used as indicators to measure the degree of chaos, nonlinear characteristics and coupling between nonlinear systems. By coupling each fault system (or chaotic system) with the healthy system (or non-chaotic system), we can identify the mode of the chaotic system and the nonlinear mechanical system, dynamics inversion and fault diagnosis analysis.
(2) Von Neumann entropy and Rényi entropy, two kinds of measurement methods, can play an important role in the study of chaotic characteristics of L ü systems. These two measures can provide a standard measure of coupling of nonlinear systems and accurately judge the state of the chaotic system.
(3) When using the full vector multi-scale Rényi entropy to study the coupling relationship between the vibration signals of the planetary gear transmission system in different states, there is a certain rule. With the change of Rényi entropy scale parameter, the positive and negative of entropy will change, which is just the expression of different physical meaning, and does not affect the problem of mode identification, dynamic inversion and fault diagnosis of nonlinear mechanical system; When the Rényi entropy scale parameter τ→5 the Rényi entropy of the multi-stage planetary gear transmission system will converge to a fixed value.
(4) According to the characteristics of low signal-to-noise ratio of mechanical system signal, the noise in vibration signal is reduced by using the compression sensing technology. The experimental signal processing results show that the compression sensing technology has good noise reduction ability and noise robustness.
(5) When the running state of the mechanical system is disturbed by noise, through the calculation of Von Neumann entropy and multi-scale Rényi entropy, the dynamics characteristics of the system are the same, that is, Von Neumann entropy and multi-scale Rényi entropy are robust to the change of the running state of the mechanical system.
